# 2449. Attributable mortality and cause of death of *Candida auris* cases, Los Angeles County

**DOI:** 10.1093/ofid/ofad500.2067

**Published:** 2023-11-27

**Authors:** Kelsey OYong, Wendy Knight, Jennifer Nguyen, Sandeep Bhaurla, Roxanne Archer, Zachary Rubin

**Affiliations:** Los Angeles County Department of Public Health, Los Angeles, CA; Los Angeles County Department of Public Health, Los Angeles, CA; Los Angeles County Department of Public Health, Los Angeles, CA; Los Angeles County Department of Public Health, Los Angeles, CA; Los Angeles County Department of Public Health, Los Angeles, CA; Los Angeles County Department of Public Health, Los Angeles, CA

## Abstract

**Background:**

*Candida auris* is a drug-resistant nosocomial pathogen that has a reported mortality rate of 30-50% in infected persons. Mortality among colonized cases has rarely been characterized. The Los Angeles County Department of Public Health (LACDPH) sought to determine attributable mortality among *C. auris* cases with specimens from sterile and non-sterile body sites.

**Methods:**

*C. auris* is a reportable condition to LACDPH, and laboratory and epidemiological factors are collected for each case. Sterile body sites included blood, surgical wound, bone, and peritoneal, ascitic, body, and bile fluids. All other body sites, including skin swabs, were categorized as non-sterile. Case data were linked with California death registry data by name and date of birth. *C. auris*-attributable deaths included any death where the terms ‘*Candida auris*,’ ‘fungemia,’ ‘Candidemia,’ ‘Candidal sepsis,’ ‘*Candida* urinary tract infection,’ ‘*Candida* endocarditis,’ or ‘*Candida* septic shock’ were listed as the immediate cause of death (COD), as a condition leading to COD, and/or listed in significant conditions contributing to death. Thirty-day mortality was defined as a death within 30 days of the first positive specimen collection.

**Results:**

Among 2,055 reported *C. auris* cases between January 2020 and March 2023, 1,080 matched a death certificate (53%); 74 deaths were cases with sterile site specimens and 970 deaths were cases with non-sterile site specimens. Thirty-day mortality varied by cases with sterile site specimens vs. non-sterile sites (26% vs. 20%, respectively), as did 30-day attributable mortality (17% vs. 1.9%, respectively). Three cases had *C. auris* listed as an immediate COD, all of which were cases with non-sterile specimens. The most common COD among cases were cardiopulmonary arrest, pneumonia, and respiratory failure.

**Conclusion:**

Attributable mortality among *C. auris* cases with sterile site specimens was lower than previously reported, possibly because the circulating clade in LAC is relatively drug-susceptible. Attributable mortality among cases with non-sterile site specimens, including skin swabs, was quite low. Vital records can provide a valuable supplement to traditional public health surveillance, but accuracy and completeness are subject to individual providers’ practice.

**Disclosures:**

**All Authors**: No reported disclosures

